# Supplementary material for: Cul5Wsb2 uses BCL2 proteins as co-receptors to target Bim for degradation
Source: bioRxiv. 2025 Oct 11:2025.08.14.670414. Originally published 2025 Aug 14. Preprint. [Version 2] doi: 10.1101/2025.08.14.670414 (PMC12363959; doi:10.1101/2025.08.14.670414)

A

Wsb2-01 ICE analysis

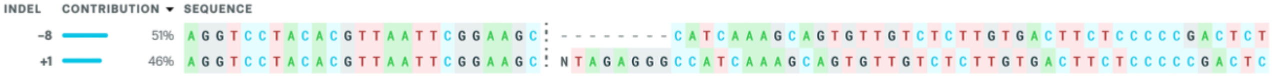

B

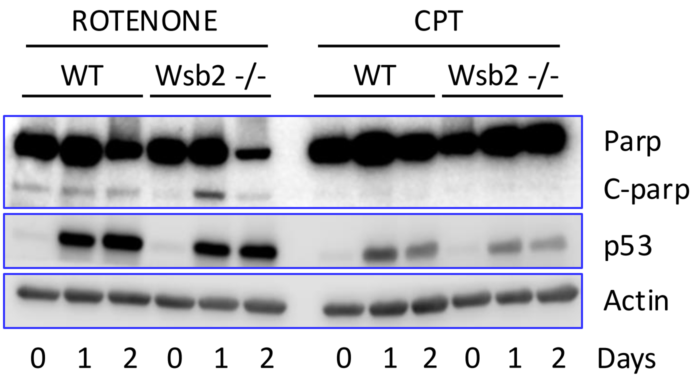

A

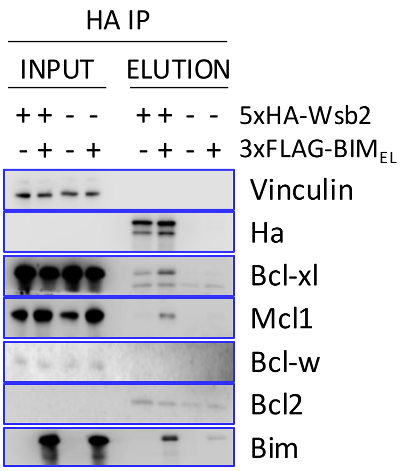
HA IP
INPUT
ELUTION
+ + - - + + - - 5xHA-Wsb2
- + - + - + - + 3xFLAG-BIM$_{EL}$
Vinculin
Ha
Bcl-xl
Mcl1
Bcl-w
Bcl2
Bim

B

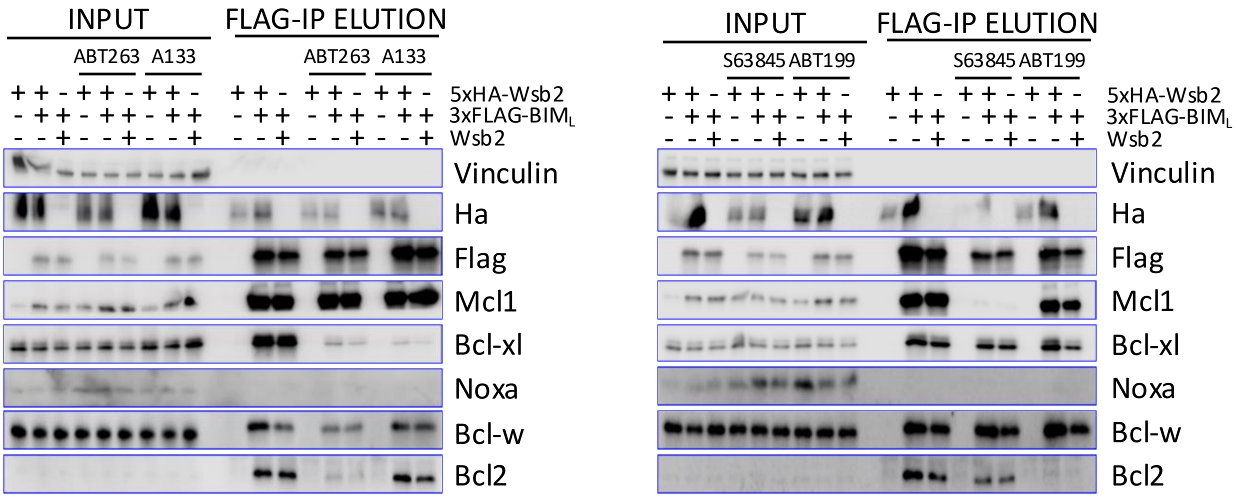
INPUT
FLAG-IP ELUTION
ABT263
A133
ABT263
A133
5xHA-Wsb2
3xFLAG-BIM$_{L}$
Wsb2
Vinculin
Ha
Flag
Mcl1
Bcl-xl
Noxa
Bcl-w
Bcl2
INPUT
FLAG-IP ELUTION
S63845
ABT199
S63845
ABT199
5xHA-Wsb2
3xFLAG-BIM$_{L}$
Wsb2
Vinculin
Ha
Flag
Mcl1
Bcl-xl
Noxa
Bcl-w
Bcl2

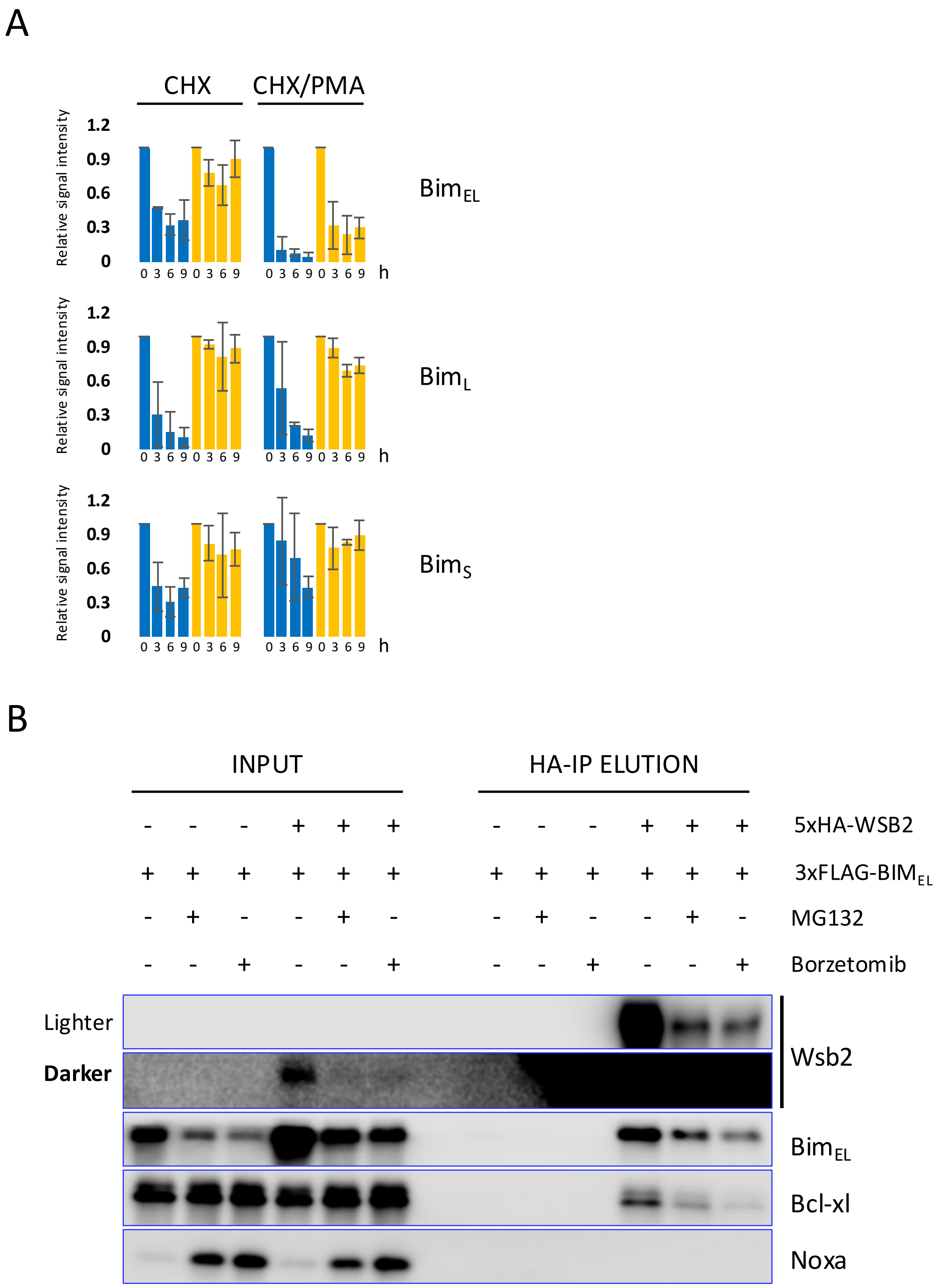
A
CHX
CHX/PMA
Relative signal intensity
1.2
0.9
0.6
0.3
0
0 3 6 9 0 3 6 9
h
Bim$_{EL}$
Bim$_{L}$
Bim$_{S}$
B
INPUT
HA-IP ELUTION
5xHA-WSB2
3xFLAG-BIM$_{EL}$
MG132
Borzetomib
Lighter
Darker
Wsb2
Bim$_{EL}$
Bcl-xl
Noxa

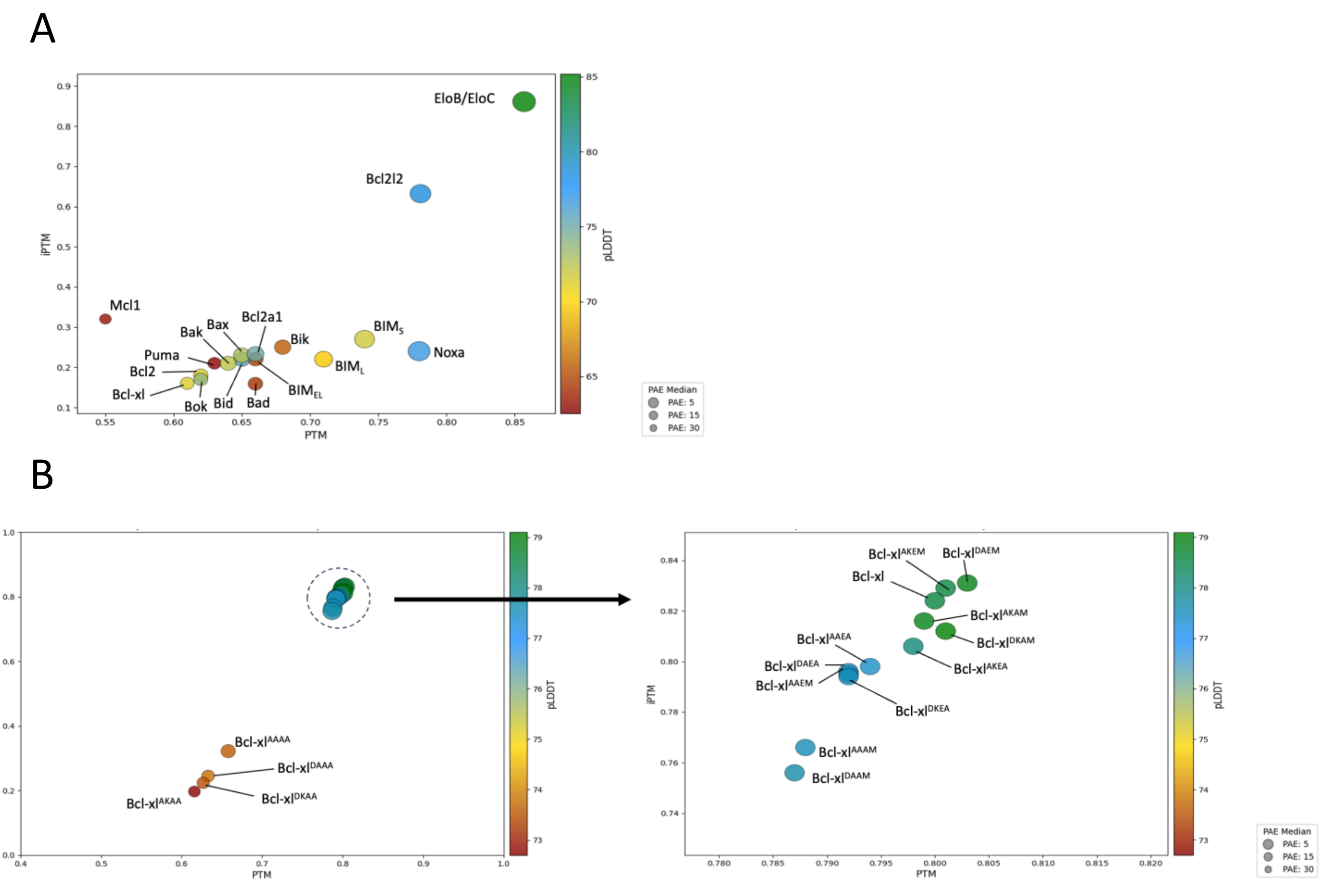

A
EloB/EloC
Bcl2l2
Mcl1
Bak
Bax
Bcl2a1
Bik
BIM$_S$
Puma
Noxa
BIM$_L$
Bcl2
Bcl-xl
Bok
Bid
Bad
BIM$_{EL}$
iPTM
PTM
pLDDT
PAE Median
PAE: 5
PAE: 15
PAE: 30
B
Bcl-xl^{AAAA}
Bcl-xl^{DAAA}
Bcl-xl^{DKAA}
Bcl-xl^{AKAA}
Bcl-xl^{AKEM}
Bcl-xl^{DAEM}
Bcl-xl
Bcl-xl^{AKAM}
Bcl-xl^{DKAM}
Bcl-xl^{AAEA}
Bcl-xl^{AKEA}
Bcl-xl^{DAEA}
Bcl-xl^{AAEM}
Bcl-xl^{DKEA}
Bcl-xl^{AAAM}
Bcl-xl^{DAAM}

A

Group	Points	Pearson	Spearman	Slope	Intercept	p-value (linregress)
Not PNS	1134	0.318	0.199	2.69E-1	-7.45E-2	4.51E-28
PNS	44	0.471	0.555	2.15E-1	-9.12E-2	1.25E-3

B

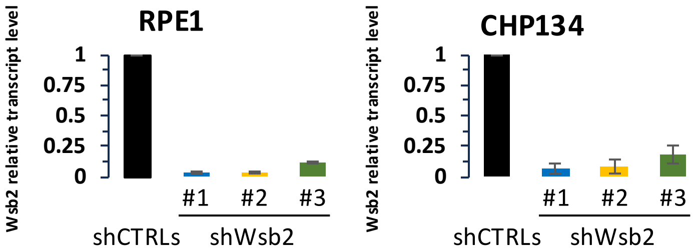

Supplement: Supplement 1 — Figure S1: Wsb2 clone sequencing validation and sensitivity to mitochondrial stressors A. ICE analysis results of Sanger sequencing results from the Wsb2 CRISPR clone generated for this study B. Immunoblot of Wild-type and Wsb2 mutant cell extract following treatment with Rotenone or Camptothecine for 72h. Figure S2: Wsb2 doesn’t directly interact with Bcl-w and Bcl2 A. As figure 2D, except cell extract were blotted for Bcl2 and Bcl-w B. Co-immunoblot analysis of 3xFLAG-BimL in the presence of the BH3 mimetics ABT-263, ABT-199, S63845, or A1331852. Immunoprecipitation of 3xFlag-Bim was used to confirm the efficacy of each BH3 mimetic in disrupting its interaction with the respective anti-apoptotic proteins. Figure S3: Quantification of CHX/PMA treatment A. Quantification of WT and Wsb2 mutant RPE1 cells from 2 immunoblots following treatment with cycloheximide with or without PMA added to one set of samples at the same time as cycloheximide. Signals were normalized to vinculin loading control and subsequently normalized to the t=0 signal. Phosphoforms of each Bim isoforms were combined for normalization. B. Western blot analysis of Borzetomib and MG132 effects on 5xHA-Wsb2 and 3xFLAG-BimEL expression compared to untreated. Figure S4: Alphafold2 MM plots A. Alphafold 2 multimer predicted Wsb1 dimerization with BCL2 protein family members scores. The Cul5 substrate adaptors EloB and EloC were used as a positive control. B. Alphafold 2 multimer predicted Wsb2 dimerization with Bcl-xl DKEM motif mutants scores. Right Panel represent a zoomed in window of the upper right cluster of predicted interaction in the left panel Figure S5: DepMap statistics & shRNA controls A. DepMap generated regression line for Peripheral Nervous System (PNS) tumors and all other tumors types (referred to as not PNS) for sensitivity to Wsb2 versus Bcl2l2 depletion B. Quantitative PCR of Wsb2 transcript levels. Constitutive expression of a Wsb2 targeting shRNA in RPE1 or CHP134 cells decrease W [file media-1.pdf]
